# Impact of the Roller Press Briquetting Process on the Morphological and Mechanical Properties of Apatite Ore

**DOI:** 10.3390/ma18071442

**Published:** 2025-03-25

**Authors:** Michał Bembenek, Vasyl Dmytriv, Łukasz Kowalski, Krzysztof Turniak, Łukasz Frocisz, Rimma Niyazbekova, Janusz Krawczyk

**Affiliations:** 1 Department of Manufacturing Systems, Faculty of Mechanical Engineering and Robotics, AGH University of Krakow, A. Mickiewicza 30, 30-059 Krakow, Poland; vasyl.t.dmytriv@lpnu.ua (V.D.); lkowalski@agh.edu.pl (Ł.K.); 2 Department of Design Machine and Automotive Engineering, Lviv Polytechnic National University, 79013 Lviv, Ukraine; 3 Institute of Geological Sciences, University of Wrocław, pl. Maksa Borna 9, 50-204 Wrocław, Poland; krzysztof.turniak@uwr.edu.pl; 4 Faculty of Metals Engineering and Industrial Computer Science, AGH University of Krakow, A. Mickiewicza 30, 30-059 Krakow, Poland; lfrocisz@agh.edu.pl; 5 Department of Standardization, Metrology and Certification, Technical Faculty, S. Seifullin Kazakh Agrotechnical Research University, 62 Zhenis Ave., Astana 010011, Kazakhstan; rimma.n60@mail.ru; 6 TOO “Kazecoengineering”, Turan St., 50/3, 225, Astana 010000, Kazakhstan

**Keywords:** briquetting, compaction, agglomeration, phosphorite rock, apatite

## Abstract

In this study, the authors investigated the briquetting of hydroxyapatite and fluorapatite rock material and evaluated the properties of briquettes prepared in a roller press. This was conducted 10 years after the manufacturing process took place. These rocks are a primary source of the mineral phosphorus, for which demand is high, particularly in agriculture. The proper handling of the material in the industry is required due to its high environmental impact. In order to correctly identify the subject of this study, the authors analyzed its composition using energy-dispersive X-ray spectroscopy, scanning electron microscopy and polarized light microscopy. Afterwards, the authors analyzed the properties of the saddle-shaped briquettes, including their surface roughness (Ra, Rq, Rt), surface Leeb hardness distribution, porosity and density. The briquettes exhibited relatively large Ra values (mean 9.67 µm). The highest hardness was registered at the specimen center (61 HV5), whereas the lowest was at the edge (25 HV5). A high density of 2.51 g/cm^3^ was achieved in the process. It was possible to obtain saddle-shaped briquettes with reproductible properties, high density (porosity of 21%) and durability without using a binder additive. The study demonstrated that roller press briquetting can be successfully utilized as a method for compacting phosphate-bearing materials for the purpose of storage transportation and further processing.

## 1. Introduction

Apatite ore deposits can be found in many areas around the world but their composition is highly diverse, depending on the location [1,2]. Simultaneously, apatite ore is one of the main natural sources of phosphorus, and is used to obtain pure elemental phosphorus [3], as well as to manufacture mineral fertilizers. The ore also has the potential to contain rare earth elements [4,5]. Discovering sustainable, efficient and ecological methods of manufacturing phosphorus-bearing fertilizers is an important research area. In particular, due to the depletion of higher quality deposits [6,7,8], the need to increase agricultural productivity [9]. In addition, there can also be geopolitical limitations in some cases [10]. According to Grand View Research, the global phosphate fertilizer market was worth $61.63 billion in 2021, with an annual growth trend of 5.7% until 2040 [11], with some estimations predicting a 100% demand increase for phosphorus fertilizers within the next 60 years [12]. Various methods of creating slow-release fertilizers from apatites are being explored [13,14] in order to increase the availability and local absorption of the element [15], due to the environmental impact of phosphorus. Unprocessed and only mechanically processed apatites can also be used as slow-release fertilizing agents [16], however their effectiveness varies depending on the quality of the raw material [17]. The excessive use of highly water-soluble [18] phosphorus fertilizers, when only 8% of the nutrient supplement is recovered by the crops grown, can lead to the dangerous eutrophication of water bodies, as the element leaches into groundwater [19,20,21]. The area of the dead zones caused by this in the Baltic sea is estimated to be as much as 17% of its total surface [22]. One of the main reasons for the formation of harmful waste is the low quality of the feedstock, which is classified as a difficult-to-enrich raw material. Only a small amount of phosphorus fines are disposed of at enterprises within the phosphorus industry. Small fractions of less than 5 mm formed during the extraction of phosphate raw materials from the subsurface are technologically unsuitable for the production of elemental phosphorus. According to [23], the yield of the fine-grained product in mines is about 35–44%. In this respect, the full and efficient use of small fractions of phosphate raw materials extracted from mines is an important economic and environmental task. The solution to this problem is related to the improvement of existing methods of agglomerating raw materials and the involvement of small fractions of phosphate raw materials in the production of waste. One of the ways to solve the issue of using small fractions of phosphate raw materials is briquetting, which will improve technological performance during thermal processing.

An important aspect of the handling and utilization of apatite ores relates to the contaminants present in the raw material, especially its cadmium content [24,25]. The prevention of cadmium soil contamination from mineral apatite fertilizers is studied by analyzing the migration mechanisms of the element [26], or by analyzing decontamination methods of the raw material [27,28,29]. The processing of the ore in preparation for the purification and concentration of phosphate compounds includes various mechanical processing techniques, such as crushing and ball milling, which impact the mineral composition of the material [30,31]. The initial composition of the ore additionally impacts its strength, thus affecting energy consumption during grinding and crushing [32]. Most commercial fertilizer manufacturing processes also employ the granulation compaction process in drum granulators [33,34]. The phosphorite ores are ground to obtain phosphorus flour, which is used as a starting product in chemical processing [35]. Phosphorus flour is a highly dusty product that is very inconvenient to transport, store and use, and as a result, the production of phosphorus flour is currently very limited. There is a worldwide use of technology for obtaining yellow phosphorus from agglomerated phosphorite fines in ore-thermal furnaces. The use of phosphate fines, which were previously transported to the landfill and stored, is associated with the development of effective technology for sintering phosphate fines on sintering machines. As a result, the production of phosphorus flour is currently very limited. The raw materials for the agglomeration process are ore fines (concentrate) and small fractions of coke. In practice, the methods of calcination of ore fines and concentrate are used. The pelletizing method is used only for finely ground concentrate. The components of the charge are mixed, moistened and pelletized in a drum pelletizer, and then loaded onto the grate of a continuous sintering machine. The preparation for sintering is reduced to the following basic operations: preparation of an agglomeration charge, primary mixing of the charge, pelletizing of the charge on special devices called granulators. The moisture content of the crude ore varies from 1 to 6.3% and the concentrate varies from 11.7 to 13.7%.

Phosphate fines (up to 44%) which cannot be fully disposed of are stored in factory territories and are a source of dust, pollution for industrial sites, and natural effluents. Existing methods do not provide a high-quality preparation of lump phosphorites, as they have significant disadvantages: low technological performance (shaft slot and drum furnaces for heat treatment of phosphorus-produced raw materials operate in drying mode), significant dusting, unacceptable production noise and bulkiness, etc. The use of unprepared raw materials in electrothermy leads to the formation of solid, liquid and gaseous waste. This significantly reduces the technological performance and worsens the environmental situation not only in the territory of the enterprise, but also in a sizeable radius around it, thus negatively and irreversibly affecting the state of soils, agricultural land, atmosphere, hydrosphere and biosphere. The elemental phosphorus obtained from unprepared raw materials passes into sludge.

In this research, the authors evaluate the briquetting process of apatite ore as a mechanical compaction process not yet evaluated in the literature. This is helpful in terms of handling bulk apatite for transportation and logistical purposes, as well as further processing [36]. According to the data [37], the yield of the fraction of phosphorus fines in the mines is 35–44%. In this respect, utilizing of small fractions of phosphate raw materials in order to generate a more complete and rational use of its resources is an urgent task. The process is used when it is important to rationally manage and recover materials from waste, including alloy elements in the steel industry [38,39] and non-ferrous metallurgy. It can also be used when there is a need to homogenize the energetic properties of biomass [40] or fine coal fractions [41], as well as to increase the material density [42]. As a focus in this study, the authors decided to evaluate the mechanical properties of prepared apatite briquettes in addition to their mineral and chemical composition. Geometrical and mechanical properties, such as hardness, density and compressive strength are helpful in assessing quality of the briquette and the compaction process [43], and are important for further mechanical processing. Briquetting can be performed with or without binding additive, however, it will affect the product’s properties [44]. Utilizing binders might hinder moisture removal processes [45] and influence the physicochemical properties of the material [46]. Depending on the further use of prepared briquettes, the type of the material and its desired target properties, different types of briquetting equipment are used, such as, for example, piston presses, screw presses, punch presses (100–200 MPa), cylindrical presses (roller and ring types, operating up to 200 MPa) [47] and granulators that form granules by pressing the material through radial holes. Vertical hydraulic presses are used mainly for metal forming, while horizontal automatic presses can handle efficiently large volumes of waste. Roller presses, known for their simplicity and high productivity, are widely used, particularly in industries requiring continuous operation. Advances in briquetting have led to the development of a high-pressure roller press with adjustable parameters, allowing flexibility for different materials. Its continuous operation reduces energy consumption and production costs [46,47]. A significant advantage of the roller press is that its continuous operation allows for a reduction in the amount of energy required for briquette production, thus minimizing costs on a larger manufacturing scale [48,49]. Depending on the choice of tool, briquettes prepared in a roller press can adopt a geometry with or without a division plane. Briquettes without a division plane, which are so-called saddle-shaped, are used where materials being consolidated have low density, a high moisture content and hydrophobic fraction that tends to become suspended in the feeder [50]. The abovementioned advantages render roller press briquetting a suitable candidate as a processing method for bulk apatite ore handling, but might impact on the material’s composition, structure and future processability, which depend on the agglomerate’s mechanical properties. Therefore, the authors decided to analyze these factors in this study.

## 2. Materials and Methods

**Figure 1:**
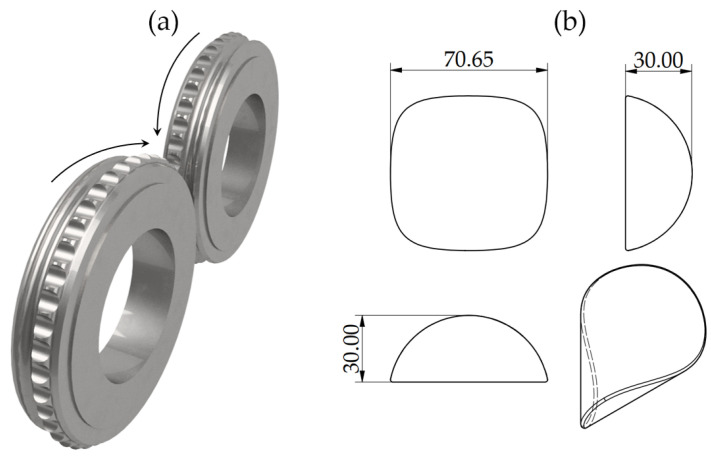
(**a**) Rolls of the compaction unit, and (**b**) theoretical shape and size of the briquette.

The material underwent the briquetting process in a LPW 450 laboratory roller press, with a gravitational feed and compaction unit (Figure 1a) for manufacturing saddle-shaped briquettes (shape and main dimensions in mm presented in Figure 1b) installed. The raw material was fed into the compacting zone with a gravitational system, with a die rotational speed of 0.1 m/s. The clearance between rotary dies was set to 1 mm. In the process, no additional binders were utilized, and the moisture content of the loose material was kept at 3%.

**Figure 2:**
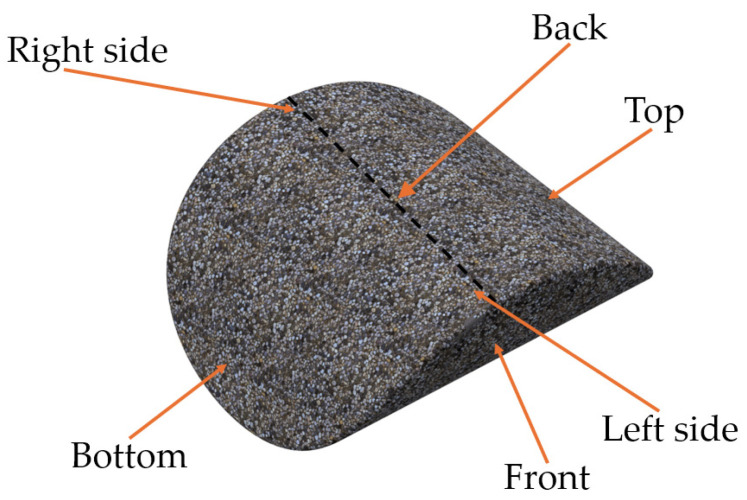
Names of the briquette’s zones analyzed in this study.

**Figure 3:**
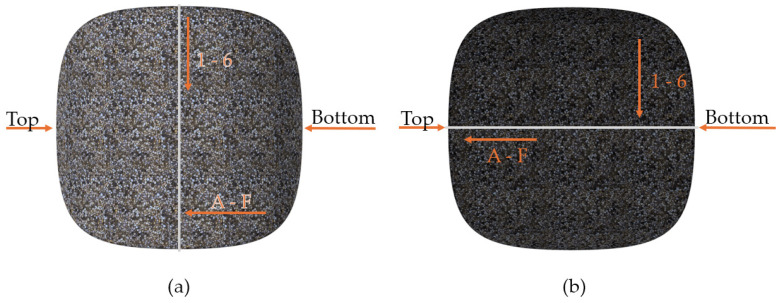
Numbering pattern for measurement fields: (**a**) front; (**b**) back. Direction of field numbering is marked by the arrows, creating an array of rows with numbers 1 to 6, and columns with symbols from A to F.

**Figure 4:**
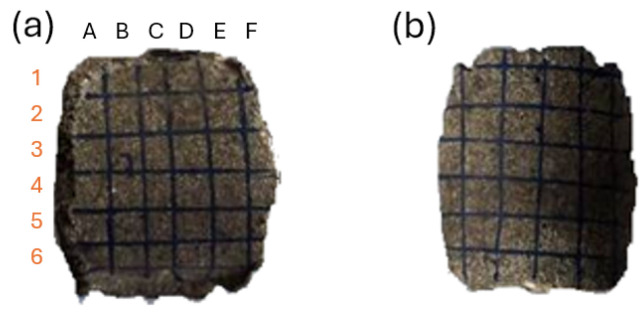
Specimen with measurement fields drawn: (**a**) front; (**b**) back. The field name on the array corresponds to column symbol and row number. For example, in drawing (**a**), the leftmost-uppermost field name is A1.

For the clarification and readability of the study, certain areas of the briquette were named, as presented in Figure 2. For hardness and roughness testing, the briquette’s areas were further divided into 72 fields (36 on the front and 36 on the back side), and were numbered in accordance with Figure 3 (rows from 1 to 6 and columns from A to F). The white line in Figure 3 represents the ridge of the briquette. The specimens with fields drawn using markers are presented in the photo (Figure 4). Each of the fields is approximately 5 by 5 mm in size.

For hardness testing, a dynamic method was selected, namely the Leeb hardness test with measurement head E. The apparatus used for the examination was Proceq EquoTip 550 (Viateco, Chorzów, Poland). The values of Leeb E hardness were recalculated automatically to Vickers HV5 hardness, with accordance with ASTM E140-12b [51]. The purpose of this test was not to obtain exact and absolute hardness values, but rather to obtain a set of comparable data between designated briquette’s zones.

The roughness tests were performed using the optical profilometer Veeco WykoNT9300 (VEECO, Los Angeles, CA, USA) in Phase Shifting Interferometry mode (PSI). For each of the marked fields, three different measurements of roughness were registered, as follows: arithmetic average of profile height deviations from the mean line (Ra), root mean square average of profile height deviations from the mean line (Rq) and total height of profile (Rt). The error induced by briquette curvature was considered omittable for the sake of this research. The roughness measurements were performed as follows:

- On the back side, in fields D1, D2, D3, D4, D5, D6 (numbering in accordance with Figure 3 and Figure 4), with the measurement path perpendicular to the top-bottom line;
- On the front side, in fields A3, B3, C3, D3, E3, F3, with the measurement path parallel to the top-bottom line.

The density of the specimen was determined using the hydrostatic method, with the following procedure:

1. Weighing of the dry briquette in the air;
2. Weighing the briquette submerged in liquid (distilled water);
3. Infusing the briquette with paraffin wax, and weighing it in the air after drying.

The utilized method allowed us to determine the bulk density and porosity degree of the material.

The samples for microscopic analysis were prepared by cutting the specimens along the division plane (gray line in Figure 3b), and then sanding the specimen’s surface with fine sandpaper to remove loose fractions from the test surface. The microstructure photographs were taken with a scanning electron microscope (Japan Electron Optics Laboratory Co., Ltd., Tokyo, Japan). Due to the large variety of particles that make up the briquette, microscopic photographs were taken in 3 different selected areas. Afterwards, in previously selected areas, the chemical elements composition was analyzed using Energy Dispersive Spectroscopy (EDS, with the following test parameters: FOV, 134 µm; voltage, 20 kV; current, 10 nA; analysis time, 60 s).

Petrographic and mineralogical studies were performed by a polarizing optical microscope (Nikon E600-Pol (Nikon, Melville, NY, USA)) and by X-ray diffraction (XRD) using the Bruker D8 Advance X-ray diffractometer (Bruker Corp., Billerica, MA, USA) at the Institute of Geological Sciences University of Wrocław, with CuKα_1,2_ operating at 40 mA and 40 kV, with a 1 mm divergence slit, 2.5° Soller slits and 5.3 mm anti-scatter slit. The sample preparation for quantitative analysis consisted of preliminary grinding using an agate pestle and mortar, and subsequent wet milling with ethanol in a McCrone Micronizing Mill with corundum grinding elements. The sample was back-loaded into the flat holder and measured using Bragg–Brentano geometry from 5 to 75° 2θ with a step size of 0.01 and 2 s counting time per step. Phase identification utilized Bruker Diffrac. Eva software (release 2019) with the ICDD PDF5+ database. Subsequently, quantitative phase analysis was performed via the full profile Rietveld method implemented in the Bruker Topas ver. 6 software. The Rietveld refinement allowed us to obtain a reliable model for the investigated XRD pattern with a R_wp_ value of 5.1.

As well as the photographs, Raman analysis tests were performed with a Renishaw inVia™ Quontor™ confocal Raman microscope (Renishaw plc, Wotton-under-Edge, UK). A LEICA DM2700M lens (Leica GmbH, Wetzlar, Germany) was used. A diode 532 nm laser was used for excitation with an emission line of a 5 mW power on the sample and a 20 s exposure time. The Raman spectra were recorded in the 200–2000 cm^−1^ range. Collected data were processed using the software Spectragryph 1.2.15. (Renishaw plc, Wotton-under-Edge, UK). Such measures were taken due to the high complexity of loose fractions of phosphorite rocks, which were utilized in the process. Such tests should also help with the replicability of the study.

## 3. Results and Discussion

**Figure 5:**
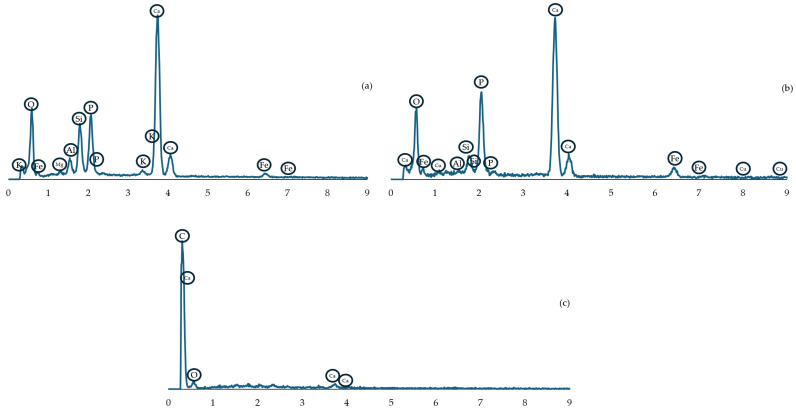
EDS spectrum graph for corresponding images in Figure 6 (graphs marked by the same (**a**–**c**) letters).

**Figure 6:**
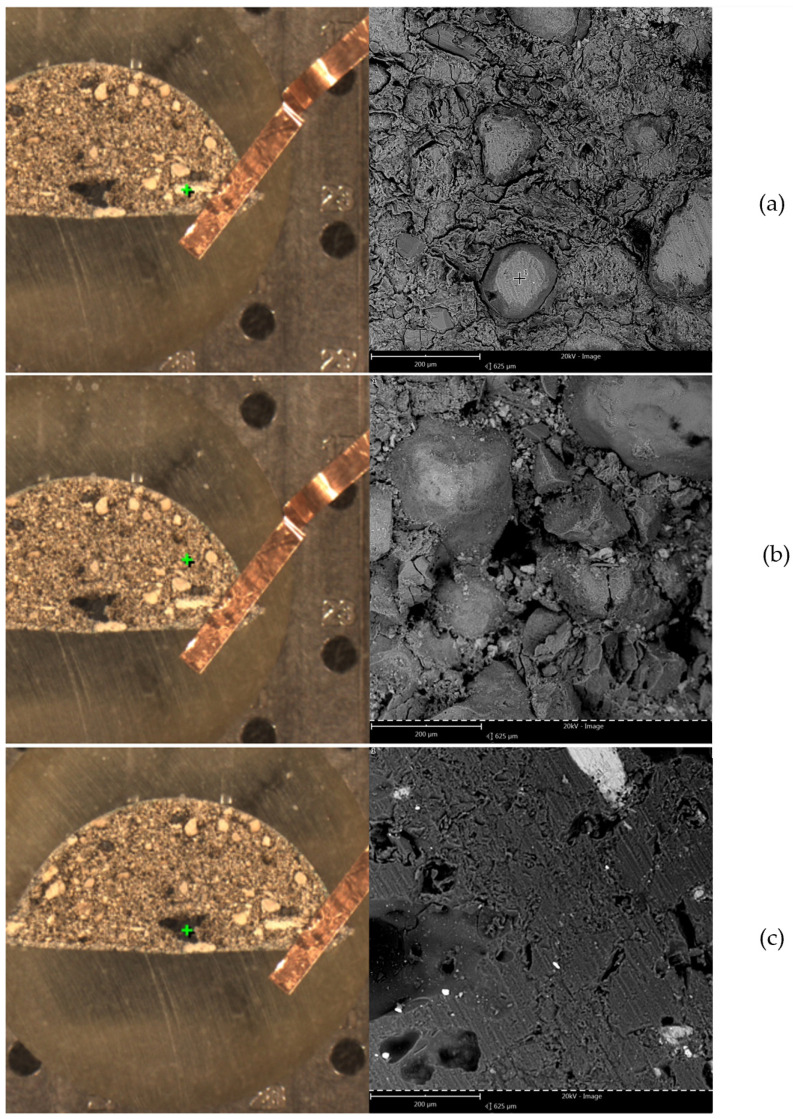
SEM microstructure images of the briquette cross-section. (**a**–**c**) represent different measurement points, marked by the green cross, and are referenced by those letters in the rest of text.

The results of the EDS analysis are presented graphically in Figure 5, where the letters (a, b, c) correspond to images in Figure 6. The EDS analysis and microstructure examination show a high variety of particles present in the briquette. The analyzed areas (marked with green cross), along with SEM photographs of the microsections, are presented in Figure 6. As is visible, most of the cross-section’s area is constituted of different powder particles and bridges created during the agglomeration process. Only the (c) view is made of uniform material mass. Similarly, the chemical composition as weight percentage is presented in Table 1. It is important to note that the results obtained might be distorted for oxygen and carbon due to the specificity of the EDS method. In areas (a) and (b), large quantities of calcium and phosphorus were observed (typical for apatite rock), along with silicon (quartz) and aluminum, possibly from potassium, calcium, magnesium or iron aluminosilicate (mineral mica). The dark spot in image (c) can be identified as a calcite grain. The porosity of the material is visible in (a), (b) and (c) in Figure 6 as well.

**Figure 7:**
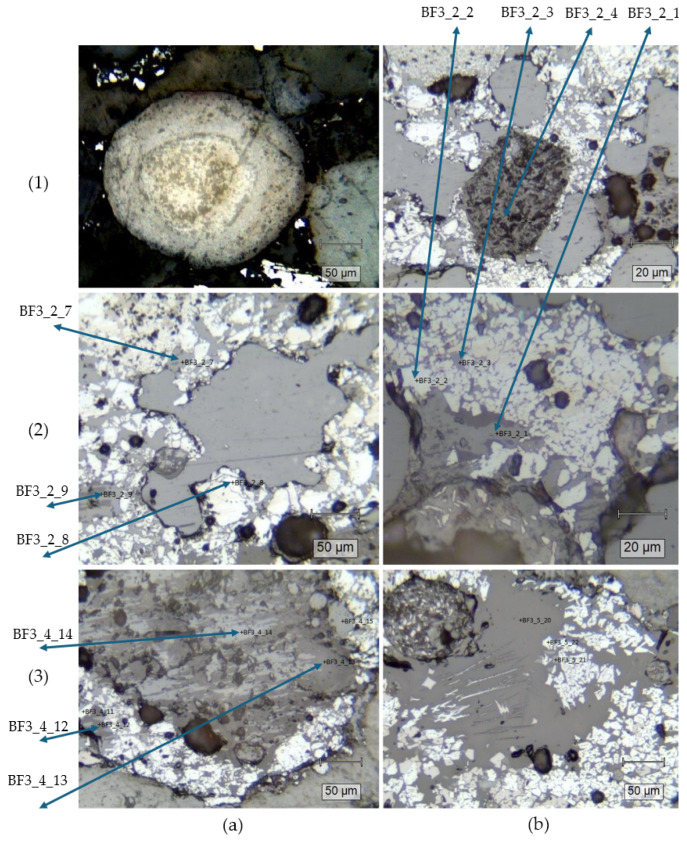
Polarizing microscope images with points of interest for Raman analysis marked.

A more detailed analysis of the materials composition and structure can be drawn from the Raman spectroscopy analysis, with some of the analyzed structures presented in polarizing microscope images (Figure 7).

In Figure 7(1a), a grain of fluorapatite was identified. The mixture also contained baryte (Figure 7(1b), BF3_2_4), hematite (Figure 7(2a), BF3_2_8) and hematite–graphite mixtures with an unidentified additive (Figure 7(2a), BF3_2_7). In Figure 7(2b) similar compounds were found, specifically hematite–graphite in BF3_2_1, BF3_2_3, and pure hematite in point BF3_2_2. In points BF3_4_12 Figure 7(3a) hematite, graphite and amphibole were identified, and more silicate crystals were present in points BF3_4_13 and BF3_4_14. Amphibole, wustite and lepidocrite could be observed in the points presented in Figure 7(3b).

**Figure 8:**
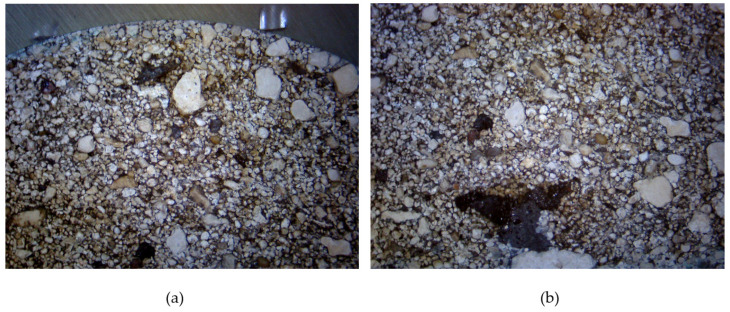
Macroscopic photograph of the briquette cross-section: (**a**) top-central area; (**b**) bottom central area.

The macroscopic structure of the briquette cross-section is visible in Figure 8. The grain size varies significantly, but the compaction process ensures an even and correct distribution.

**Figure 9:**
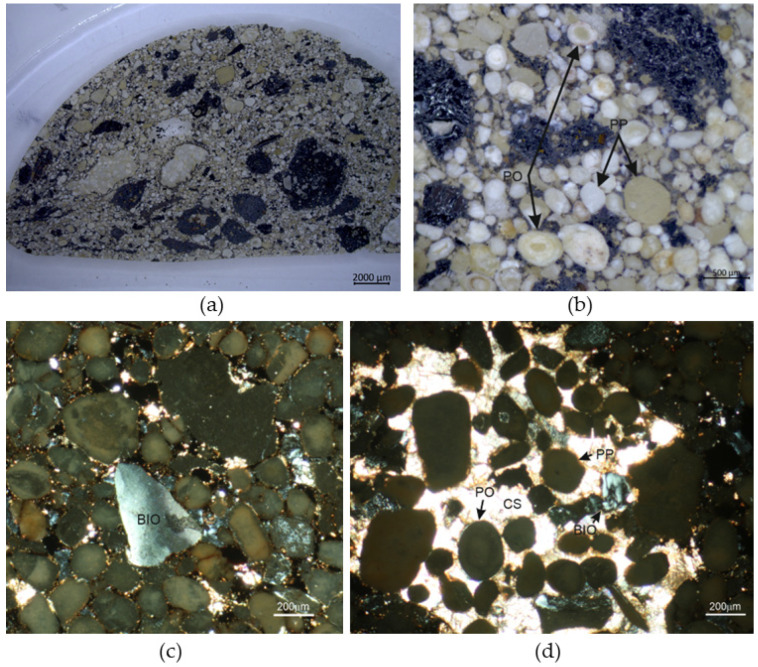
(**a**) Cross-section of the briquette; (**b**) phosphatic ooid (PO) and phosphatic pellet in the briquette; (**c**) bio-fragment (BIO) composed of apatite; (**d**) intraclast with calcitic sparite (CS) enveloping ooids, pellets and bio-fragments. Cross-polarized with a transmitted-light micrograph.

The briquetted material can be classified petrographically as sedimentary phosphorite. The quantitative phase analysis, based on the full profile Rietveld method, shows that the briquetted material is composed mostly of carbonate fluorapatite (francolite; 83.5 wt. %), magnetite (5.2 wt. %), quartz (3.0 wt. %), calcite (2.9 wt. %), hematite (2.3 wt. %), hornblende (2.1 wt. %) and dolomite (1.0 wt. %). Texturally, the material is non-equigranular and is composed of mostly rounded light and dark grains with diameters typical of sand fraction (Figure 9a). The phosphate component occurs as ooids, pellets, bio-fragments and as a main constituent of the micritic matrix and light intraclasts.

*Ooids* are white, gray or cream-colored and have an ellipsoidal or spherical shape. They show concentric zonation with lamellas composed of mostly cryptocrystalline francolite (Figure 9b). Some ooids contain a nucleus.

*Pellets* are more diverse in shape and show a structureless interior pattern, which makes them different from ooids (Figure 9b). In addition to being ellipsoidal or spherical, they have more irregular shapes. Some pellets contain fossils and small grains of quartz as inclusions.

*Bio-fragments* include shells and other diverse skeletal elements. The mineral composing the biogenic component is optically anisotropic and shows low interference colors, which is typical of crystalline apatite (Figure 9c).

*Light intraclasts* form composite grains measuring up to 4 mm. Some of them are composed of calcitic sparite enveloping ooids, pellets, bio-fragments and Fe-oxides (Figure 9d). The others contain phosphatic ooids and pellets cemented by cryptocrystalline francolite.

The non-phosphatic grains present in the briquetted material are mostly Fe-oxides (magnetite and hematite) and quartz. In microscopic examination, scarce grains of dolomite and baryte were also detected as accessory phases. The Fe-oxides grains are opaque, dark in color and usually polymineral, showing a varying mineral composition. Some dark grains contain numerous inclusions of quartz whereas the others possess inclusions of francolite, hornblende or olivine. Quartz forms well-rounded grains ranging mostly from 0.3 to 0.5 mm (up to 0.8 mm), showing the characteristic undulose extinction of light under microscopic examination.

The results of the hardness tests of the briquetted samples are presented in Table 2 and Table 3. The obtained values show that the largest hardness for both sides was achieved in the central area of the specimen—namely 61 HV5 for 3C (back surface) and 40 HV5 for 3B (front surface). These results could be influenced by the thickness of the specimen, which is largest in the middle area, and where the density of the briquette is theoretically the highest. The lowest hardness of 25 HV5 was measured on the top area of both sides of the briquette.

**Figure 10:**
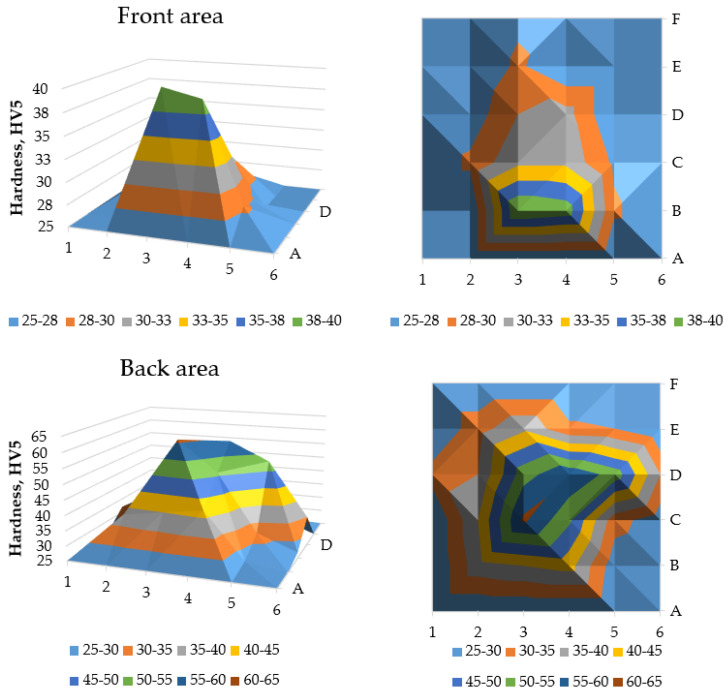
Graphical representation of the measured hardness values. Letters A–F and numbers 1–6 on graphs axes correspond to the measurement fields numbering system presented in Figure 3 and Figure 4.

Results from the abovementioned tables, for readability purposes, are presented in Figure 10. The blue color (fields close to the edge of the specimen) represents areas where measurements were not possible due to the limitations of the method. The 15 × 15 mm quasi-square field in the middle of the back of the specimen represents hardness above 40 HV5, while the comparable area in the middle of the specimen’s front area exhibited hardness close to 40 HV5. Notably, the hardest areas appear toward the end of the compaction cycle on the front, while the back has higher hardness, but is more centrally distributed. This change in hardness mirrors trends seen in other materials processed via briquetting. The highest hardness of the briquettes is observed in the central part, and then the hardness decreases towards the edge of the briquette. Similar dependencies observed for different groups of materials suggest that the design of the tools used in the process has the greatest impact on the nature of hardness changes after briquetting [52].

**Figure 11:**
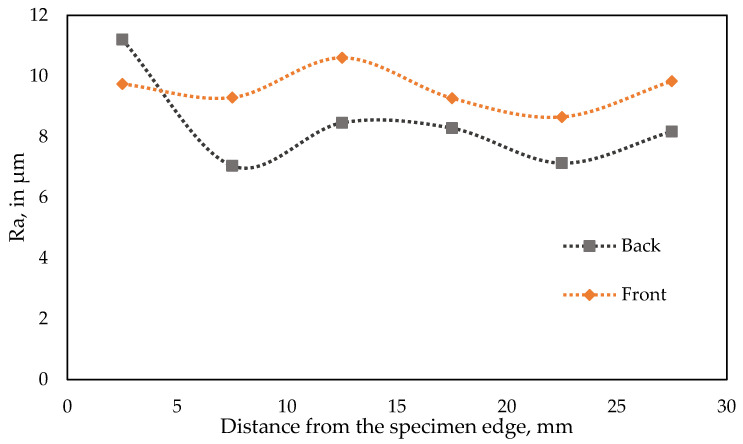
Roughness test results (Ra), graphical representation.

Detailed roughness tests results are presented in Table 4 for the back side of the briquette, and in Table 5 for the front side. For both sides, the same trend of Ra roughness was observed, in which roughness on the edges of the specimen is increased due to the material chipping in those areas. Larger roughness values were also observed in the center, likely due to the stress distribution during compaction. The high roughness value registered in field D1 (standing out in Figure 11 in comparison to front side Ra curve) is caused by the incorporation of a random abnormal material grain in this area. The briquetting of materials with different particle sizes introduces significant variability to the observed changes in roughness. When a comparison is made between the tested rock phosphate and other materials subjected to this process, it can be observed that the Ra parameter is more heterogeneous and does not correspond to the shape of the briquette. The variations in the roughness of the sample’s surface are consistent with the inhomogeneous distribution of stresses observed on the surface of these briquettes [52,53].

The values registered during the density testing of the briquette are presented in Table 6. The achieved bulk density of the specimen is influenced by its structure. The varying grain sizes of the bulk powder used in the process help to effectively fill spaces between different grains in the briquette. This effect is also visible in the microscopic photographs. A density of 79% is exceptional in terms of material agglomeration. The material’s resulting porosity, attributable to the presence of numerous particles with reduced cross-sections, is sustained at approximately 20%, which is a notably low value for briquettes produced by this method. In the case of metallic materials, porosity alterations are found to be profoundly contingent on the briquette’s location, exhibiting a range from approximately 50% to 14%, contingent on the specific location. In the case of the material under investigation, the porosity was more evenly distributed and it related to the spaces between the finest compacted particles [52,53].

**Figure 12:**
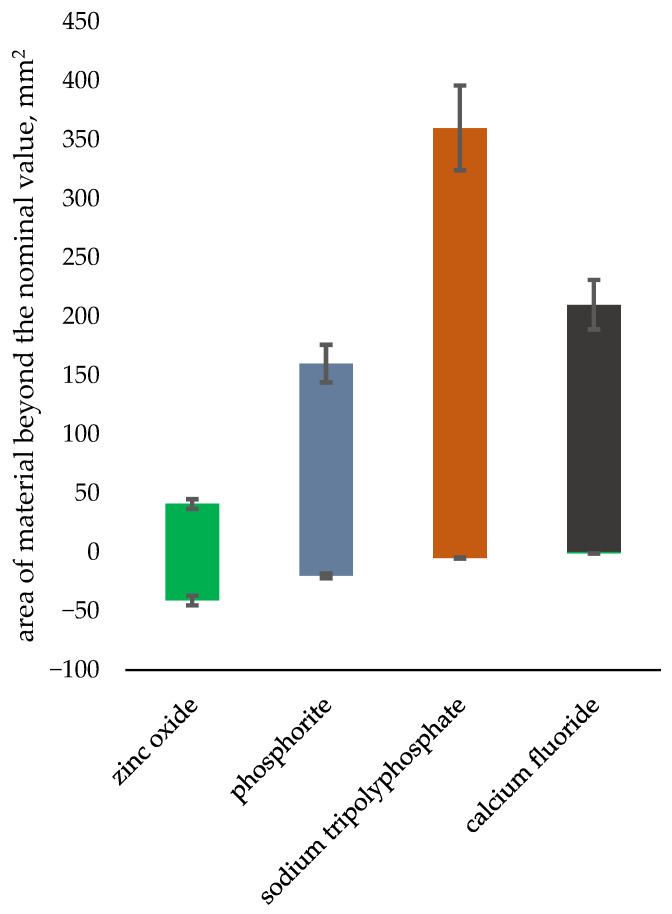
Comparison of the tested phosphorite with other ceramic materials in terms of dimensional inaccuracy.

A comparative dimensional analysis was performed, in relation to other materials analyzed by the authors [54]. The area of material above the nominal value is the mean area of cavities and chipping occurring during the briquetting process, and the area below the nominal value is the area of surplus material in comparison to other ceramic materials. The analysis results are presented in Figure 12. Above average chipping was observed for the briquetted apatite, which can be explained by the varying size of bulk particles undergoing compression.

**Figure 13:**
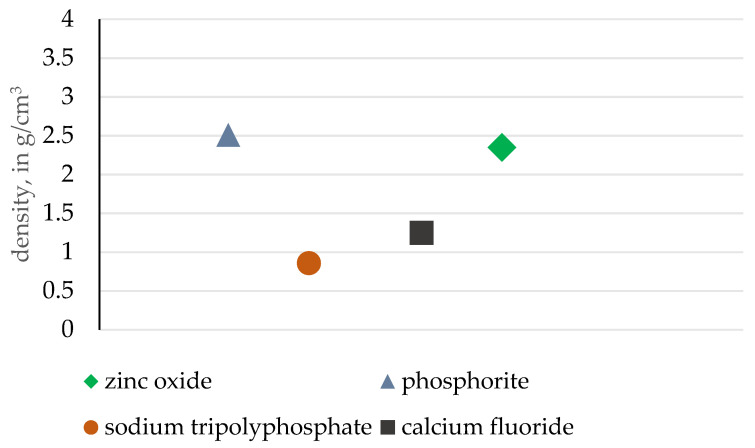
Comparison of the tested phosphorite with other ceramic materials in terms of density.

A similar density comparison is presented in Figure 13. The phosphorite ore analyzed is characterized by the highest density value, while the briquetting pressure is the lowest among compared materials, signifying the high affinity of apatite ore for roller press briquetting. Once again, such an observation can be expected due to the high variety of bulk particle sizes, as smaller particles can fill the gaps between larger crystallites. The barite inclusions observed also positively influence the density factor.

## 4. Conclusions

Processes of phosphorite ore briquetting are under-researched in the literature, but economically important (being non-renewable and main source for phosphorus) and environmentally significant. They can help decrease the phosphorus pollution created during processing and transporting and can be utilized as an alternative to other compaction methods or to prepare slow-release, low-cost apatite fertilizers. This research has proved that it is possible to manufacture saddle-shaped phosphorite briquettes with desirable properties using a roller press, without adding bonding agents to the material. The high material density achieved shows it has good affinity with the briquetting process. The high density is achieved due to the variety of different mineral particles with varying sizes. Roughness measurements point to higher material density in the bottom area, which is typical for briquettes manufactured on roller presses in general. The material composition, high amounts of apatite, quartz and mica, point to its origin in the Russian craton. Phosphorite can be considered a material with high suitability for compaction processes, even in comparison to different materials for which pressure agglomeration processes are commonly used. The results presented in [52,53] support the observation of the highest density and stress concentration in the top area of the briquette, which is characteristic of manufacturing saddle-shaped briquettes in a roller press. This observation points to the correctness of the selected shape and agglomeration method. The obtained shape and properties of the agglomerate were repeatable among multiple specimens, despite the complexity of the bulk material. The results prove that filler material is not required for the selected briquetting process, so in effect, the composition of the bulk and the agglomerate remains the same for future processing purposes.

## Figures and Tables

**Table 1:** Chemical composition of tested specimens in corresponding areas.

Element Symbol	Weight %		
	(a) ^1^	(b) ^1^	(c) ^1^
O	54.3	55.7	45.1
Ca	27.0	26.6	4.1
P	8.4	11.4	-
Si	5.4	2.0	-
Al	2.3	0.2	-
Fe	1.4	3.8	-
K	0.8	-	-
Mg	0.4	-	-
Cu	-	0.3	-
C	-	-	50.8

^1^ The letters correspond to analysis points a, b and c presented in Figure 6.

**Table 2:** Hardness test results for back surface of the specimen (HV5).

		1	2	3	4	5	6
Bottom	A	x	x	x	x	x	x
	B	x	38	40	42	27	x
	C	25	40	61	51	32	27
	D	32	35	53	59	53	35
	E	x	33	39	31	30	x
Top	F	x	x	x	x	x	x

The letters A–F and numbers 1–6 correspond to the measurement fields numbering system presented in Figure 3 and Figure 4. Fields without a measured value are marked by x.

**Table 3:** Hardness test results for the front surface of the specimen (HV5).

		1	2	3	4	5	6
Bottom	A	x	x	x	x	x	x
	B	x	x	40	39	28	x
	C	x	28	32	32	27	x
	D	x	26	30	31	x	x
	E	x	x	28	25	x	x
Top	F	x	x	27	26	x	x

The letters A–F and numbers 1–6 correspond to the measurement fields numbering system presented in Figure 3 and Figure 4. Fields without a measured value are marked by x.

**Table 4:** Measured roughness values on the back side of the briquette.

Field Symbol	D1	D2	D3	D4	D5	D6
Ra, in µm	11.20	7.04	8.46	8.28	7.13	8.17
Rq, in µm	15.33	8.88	10.87	10.41	9.38	10.39
Rt, in µm	136.77	115.18	117.21	121.53	143.81	130.84

The symbols D1–D6 correspond to the measurement fields numbering system presented in Figure 3 and Figure 4.

**Table 5:** Measured roughness values on front side of the briquette.

Field Symbol	A3	B3	C3	D3	E3	F3
Ra, in µm	9.74	9.29	10.60	9.27	8.65	9.83
Rq, in µm	12.27	11.15	12.86	11.37	11.05	12.74
Rt, in µm	164.46	86.62	141.08	100.04	147.46	126.20

The symbols A3–F3 correspond to the measurement fields numbering system presented in Figure 3 and Figure 4.

**Table 6:** Results of density tests.

Weight of the dry briquette, in g	20.1265
Weight of paraffin infused briquette, in g	20.7392
Weight of the briquette in distilled water, in g	12.7583
Bulk density, in g/cm^3^	2.51
Porosity, in %	21

## Data Availability

The original contributions presented in the study are included in the article, further inquiries can be directed to the corresponding author.

## References

[1] Mao M., Rukhlov A.S., Rowins S.M., Spence J., Coogan L.A. (2016). Apatite Trace Element Compositions: A Robust New Tool for Mineral Exploration*. Econ. Geol..

[2] O’Sullivan G., Chew D., Kenny G., Henrichs I., Mulligan D. (2020). The Trace Element Composition of Apatite and Its Application to Detrital Provenance Studies. Earth-Sci. Rev..

[3] Orabi A., El-Sheikh E., Hassanin M., El Kady M., Abdel-Khalek M., Mowafy A. (2018). Extraction of Rare Earth Elements from Abu–Tartour Wet Process Phosphoric Acid Using Synthesized Salicylaldehyde Azine. Miner. Eng..

[4] Ihlen P.M., Schiellerup H., Gautneb H., Skår Ø. (2014). Characterization of Apatite Resources in Norway and Their REE Potential—A Review. Ore Geol. Rev..

[5] Ramos S.J., Dinali G.S., de Carvalho T.S., Chaves L.C., Siqueira J.O., Guilherme L.R.G. (2016). Rare Earth Elements in Raw Materials and Products of the Phosphate Fertilizer Industry in South America: Content, Signature, and Crystalline Phases. J. Geochem. Explor..

[6] Yang B., Cao S., Zhu Z., Yin W., Sheng Q., Sun H., Yao J., Chen K. (2021). Selective Flotation Separation of Apatite from Dolomite Utilizing a Novel Eco-Friendly and Efficient Depressant for Sustainable Manufacturing of Phosphate Fertilizer. J. Clean. Prod..

[7] Ryszko U., Rusek P., Kołodyńska D. (2023). Quality of Phosphate Rocks from Various Deposits Used in Wet Phosphoric Acid and P-Fertilizer Production. Materials.

[8] Dahanayake K., Ratnayake M.P.K., Sunil P.A. (1995). Potential of Eppawala Apatite as a Directly Applied Low-Cost Fertilizer for Rice Production in Sri Lanka. Fertil. Res..

[9] Giyasidinov A.L., Sultonov B.E., Dormeshkin O. (2023). Study on the Composition of Phosphorus Fertilizers Obtained on the Basis of Kizilkum Phosphorites and Nitric Acid. J. Chem. Technol. Metall..

[10] Syvyi M., Demyanchuk P., Havryshok B., Zablotskyi B. (2023). Phosphates of Ukraine as raw materials for the production of mineral fertilizers and ameliorants. Gospod. Surowcami Miner.—Miner. Resour. Manag..

[11] Phosphate Fertilizer Market Size & Share Report, 2040. https://www.grandviewresearch.com/industry-analysis/phosphate-fertilizers-market.

[12] Cordell D., White S. (2014). Life’s Bottleneck: Sustaining the World’s Phosphorus for a Food Secure Future. Annu. Rev. Environ. Resour..

[13] Chen M., Li Z., Huang P., Li X., Qu J., Yuan W., Zhang Q. (2018). Mechanochemical Transformation of Apatite to Phosphoric Slow-Release Fertilizer and Soluble Phosphate. Process Saf. Environ. Prot..

[14] de Morais E.G., Silva C.A. (2023). Novel Slow-Release NPK Biochar-Based Fertilizers with Acidulated Apatite: Evaluation of the Fertilization Value in a Short-Term Experiment. J. Soil Sci. Plant Nutr..

[15] Timofeeva T.A., Chebotar V.K., Demidov D.V., Gaidukova S.E., Yakovleva I.V., Kamionskaya A.M. (2023). Effects of Apatite Concentrate in Combination with Phosphate-Solubilizing Microorganisms on the Yield of Ryegrass Cultivar Izorskiy. Agronomy.

[16] Aarnio T., Räty M., Martikainen P.J. (2003). Long-Term Availability of Nutients in Forest Soil Derived from Fast- and Slow-Release Fertilizers. Plant Soil.

[17] Hackman J., Ozyhar T., Chien S.H., Hilty F., Woodley A., Cook R.L. (2022). Evaluation of Synthetic Hydroxyapatite as a Potential Phosphorus Fertilizer for Application in Forest Plantations. For. Sci. Technol..

[18] Tennakone K., Weerasooriya S.V.R., Jayatissa D.L., Damayanthi M.L.W.D., Silva L.H.K. (1988). Non Hygroscopic Superphosphate Fertilizer from Apatite and Hydrochloric Acid. Fertil. Res..

[19] Review of AHDB-Funded Research on Phosphorus Management in Arable Crops|AHDB. https://ahdb.org.uk/review-of-ahdb-funded-research-on-phosphorus-management-in-arable-crops.

[20] Conley D.J., Paerl H.W., Howarth R.W., Boesch D.F., Seitzinger S.P., Havens K.E., Lancelot C., Likens G.E. (2009). Controlling Eutrophication: Nitrogen and Phosphorus. Science.

[21] Blackwell M.S.A., Darch T., Haslam R.P. (2019). Phosphorus Use Efficiency and Fertilizers: Future Opportunities for Improvements. Front. Agric. Sci. Eng.—FASE.

[22] Smith V.H. (2003). Eutrophication of Freshwater and Coastal Marine Ecosystems a Global Problem. Environ. Sci. Pollut. Res..

[23] Altybaev J.M. (2013). Scientific and Technological Bases of Obtaining Ni-Co Containing Agglomerates in Phosphorus Production. Ph.D. Thesis.

[24] Mar S.S., Okazaki M. (2012). Investigation of Cd Contents in Several Phosphate Rocks Used for the Production of Fertilizer. Microchem. J..

[25] Iretskaya S.N., Chien S.H., Menon R.G. (1998). Effect of Acidulation of High Cadmium Containing Phosphate Rocks on Cadmium Uptake by Upland Rice. Plant Soil.

[26] Zou C., Shi Z., Yang Y., Zhang J., Hou Y., Zhang N. (2023). The Characteristics, Enrichment, and Migration Mechanism of Cadmium in Phosphate Rock and Phosphogypsum of the Qingping Phosphate Deposit, Southwest China. Minerals.

[27] Zalim Y., Benayada A., El Ahmadi Z. (2022). Cadmium Removal from Cadmium-Containing Apatites by Ion-Exchange Reactions. ChemistrySelect.

[28] Santos I.D., Rodrigues S.L., Siqueira J.O., Monte M.B.M., Dutra A.J.B. (2016). Effect of Partial Oxidation of Organic Matter on Cadmium Leaching from Phosphate. Miner. Eng..

[29] Benredjem Z., Delimi R., Khelalfa A. (2012). Phosphate Ore Washing by Na_2_ EDTA for Cadmium Removal: Optimization of the Operating Conditions. Pol. J. Chem. Technol..

[30] Fan Y., Zhang G., Li S., Zhang L., Guo J., Feng C. (2024). Mineral Liberation and Concentration Characteristics of Apatite Comminuted by High-Pressure GRU. Minerals.

[31] Al-Wakeel M.I. (2005). Effect of Mechanical Treatment on the Mineralogical Constituents of Abu-Tartour Phosphate Ore, Egypt. Int. J. Miner. Process..

[32] Neradovsky Y.N., Kompanchenko A.A., Chernyavsky A.V. (2022). Texture and Mineral Composition of Magmatic Apatite-Nepheline Ores: Technological Consequences (Exemplified by Khibiny). IOP Conf. Ser. Earth Environ. Sci..

[33] (2000). Best Available Techniques for Pollution Prevention and Control in the European Fertilizer Industry, Booklet No. 7 of 8: Production of NPK Compound Fertilizers by Nitrophosphate Route.

[34] Abouzeid A.-Z.M. (2008). Physical and Thermal Treatment of Phosphate Ores—An Overview. Int. J. Miner. Process..

[35] Zhantasov K.T., Bazhirova K.N., Toltebayeva Z.D., Zhantasova D.M. (2013). Current Status, Problems and Prospects of Development of Phosphate Industry in Khazakhstan. Chem. Ind. Today.

[36] Kaliyan N., Morey R.V. (2010). Natural Binders and Solid Bridge Type Binding Mechanisms in Briquettes and Pellets Made from Corn Stover and Switchgrass. Bioresour. Technol..

[37] Tleuov A.S. (2015). Waste Disposal of Enterprises of the Phosphorus Industry. Textbook.

[38] Magdziarz A., Kuźnia M., Bembenek M., Gara P., Hryniewicz M. (2015). Briquetting of EAF Dust for Its Utilisation in Metallurgical Processes. Chem. Process Eng..

[39] Zhang H., Hui L., Dong J., Xiong H. (2018). Optimization of the Stainless Steel Dust Briquette Reduction Process for Iron, Chromium, and Nickel Recovery. High Temp. Mater. Process..

[40] Grover P.D., Mishra S.K. (1996). Biomass Briquetting: Technology and Practices.

[41] Bembenek M., Dzik T., Smyła J., Kozłowski A., Wojtas P. (2022). Comparative Analysis of Combustion of Qualified Composite Fuel for the Transitional Period in the Household and Communal Sector in Poland. Manag. Syst. Prod. Eng..

[42] Kaur A., Roy M., Kundu K. (2017). Densification of Biomass by Briquetting: A Review. Int. J. Recent Sci. Res..

[43] Mitchual S.J., Katamani P., Afrifa K.A. (2019). Fuel Characteristics of Binder Free Briquettes Made at Room Temperature from Blends of Oil Palm Mesocarp Fibre and Ceiba Pentandra. Biomass Convers. Biorefinery.

[44] Obi O.F., Pecenka R., Clifford M.J. (2022). A Review of Biomass Briquette Binders and Quality Parameters. Energies.

[45] Xu B., Chu M., Hong A.B., Zhang F.L. (2013). Comparative Study on Pyrolysis Characteristics of Lignite Binderless Briquette and Raw Coal. Appl. Mech. Mater..

[46] Olugbade T., Ojo O., Mohammed T. (2019). Influence of Binders on Combustion Properties of Biomass Briquettes: A Recent Review. BioEnergy Res..

[47] Borowski G. (2011). Wykorzystanie Brykietowania Do Zagospodarowania Odpadów.

[48] Michrafy A., Zavaliangos A., Cunningham J.C., Pandey P., Bharadwaj R. (2017). 4—Dry Granulation Process Modeling. Predictive Modeling of Pharmaceutical Unit Operations.

[49] Bembenek M. (2017). Badania i Perspektywy Nowych Obszarów Stosowania Pras Walcowych. Przem. Chem..

[50] Bembenek M., Uhryński A. (2021). Analysis of the Temperature Distribution on the Surface of Saddle-Shaped Briquettes Consolidated in the Roller Press. Materials.

[51] (2019). Hardness Conversion Tables for Metals Relationship Among Brinell Hardness, Vickers Hardness, Rockwell Hardness, Superficial Hardness, Knoop Hardness, Scleroscope Hardness, and Leeb Hardness.

[52] Bembenek M., Krawczyk J., Frocisz Ł., Śleboda T. (2021). The Analysis of the Morphology of the Saddle-Shaped Bronze Chips Briquettes Produced in the Roller Press. Materials.

[53] Bembenek M., Buczak M., Baiul K. (2022). Modelling of the Fine-Grained Materials Briquetting Process in a Roller Press with the Discrete Element Method. Materials.

[54] Zięba A. (2019). Influence of the Type of Briquetting Material on the Morphology of the Saddle-Shaped Briquette. Master’s Thesis.

